# Challenges and opportunities for greater public-private partnership for the implementation of the WHO operational framework for building climate resilient health systems to improve malaria control and elimination in Sub-Saharan Africa: a rapid review

**DOI:** 10.3389/frhs.2025.1593923

**Published:** 2025-09-09

**Authors:** Sheila Lumumba, Samuel Kamau, Isaac Ntwiga, Josphat Martin Muchangi, Jackline Kiarie, Sarah Kosgei, Moses Mwamburi, George Kimathi

**Affiliations:** ^1^ Institute of Capacity Development, Amref Health Africa, Nairobi, Kenya; ^2^ Reinit Research Limited, Nairobi, Kenya; ^3^ Amref Health Africa, Nairobi, Kenya

**Keywords:** public-private partnerships, malaria, climate change, implementation, WHO framework

## Abstract

The relationship between climate change and malaria is complex, with both predictable and unpredictable aspects. The impacts of climate change may promote mosquito breeding, increase parasite development rates and extend the geographical range of malaria vectors through increased temperature and rainfall. In addition, climate change influences the transmission of malaria indirectly through social and economic pathways. The gains made in malaria control are evidently under threat. Partnerships to build climate resilient health systems for malaria control in Sub-Saharan Africa (SSA) should be harnessed to implement the WHO Operational Framework in SSA. This review follows the Preferred Reporting Items for Systematic Reviews and Meta-Analyses (PRISMA) and the Centre for Reviews and Dissemination (CRD) guidelines. A search strategy was formulated based on the PECOS framework using BOOLEAN operators “AND” and “OR” for all possible combinations of the following search teams: public-private sector partnerships, public health, and Sub-Saharan Africa. We identified 173 research papers from our database searches, and this systematic review includes 11 articles focusing on the objective of this study. The included studies identified challenges such as ineffective legal and policy frameworks, bureaucracy, limited buy-in and adherence to guidelines by private partners and a lack of systemic integration of climate risk assessments in health planning among others. On the other hand, opportunities lie within the health workforce, essential medicines and technologies, and emergency preparedness and management. These include health workforce education and training through massive open online courses, proper response targeting in partnership with the private sector, and co-production mechanisms for climate change and malaria research. PPPs remain a viable alternative in the adaptation of the WHO Operational Framework despite the challenges they face. This is particularly the case when the technical and financial capacities of the countries in the SSA region are considered. There are lessons to be derived and best practices to be instituted from case studies of previous partnerships, especially in malaria control.

## 1 Introduction

The relationship between climate change and malaria is complex, with both predictable and unpredictable aspects. Malaria is related to socio-economic and environmental factors across multiple sectors. A systematic review found a 20% reduction in malaria infection odds per unit increase in the wealth index (1). These differences may reflect disparities in access to prevention measures or even diagnosis and treatment-seeking behaviors between different SES levels. Factors such as quality of housing are linked to malaria incidence because many mosquito bites in parts of Africa result from mosquitoes resting indoors (2–4). Occupation can also be directly linked to malaria incidence by increasing contact with vectors, such as in agricultural settings with irrigation pools, fish farming, and nomadic pastoralism.

According to Pardy et al., climate change exacerbates wealth inequality globally and at sub-national scales (5). Inequality is magnified through reduced economic activity, asset degradation, and limited healthcare access, especially in underdeveloped, agriculture-dependent rural areas. Sub-Saharan Africa is especially vulnerable to this since a majority of the population in SSA live in rural areas (6) and engage in agriculture as their main economic activity (7). This means that the effects of climate change on the socio-economic status of populations will spill over into malaria control efforts.

We must build health systems that adapt to environmental changes for sustained and improved malaria surveillance, prevention and treatment. This will require an all-hands-on-deck approach to climate-resilient health systems, specifically malaria control efforts. In their guide to measuring the climate resilience of health systems, the WHO cites the need to keep up with upstream determinants of exposure and vulnerability, that arise from both health and non-health sectors. It also calls for engagements with the key public and private stakeholders in both sectors (8). The World Bank defines a public-private partnership (PPP) as “*A long-term contract between a private party and a government entity, for providing a public asset or service, in which the private party bears significant risk and management responsibility, and remuneration is linked to performance”* (9).

These partnerships have been effective in health system-strengthening activities in Sub-Saharan Africa and beyond. South Africa has extensive experience establishing PPPs, leading to physical infrastructure upgrades, improved healthcare quality, and better recruitment and retention of scarce skills (10). The Emergency Hiring program in Kenya was established to address the health workforce shortage in a period when the health system was under pressure from HIV/AIDs patients. The led to an increase in doctors (from 1.78–1.89) and nurses and midwives (from 3.81–8.22) per 10,000 people (11). In Nigeria, national mental health policies were successfully merged with WHO guidelines through a PPP, improving service delivery (12). In Ghana, Non-Governmental and Faith-Based Organizations participation led to an increase in the range of healthcare services to underserved communities and geographical areas, covering gaps in the public health agenda (13).

Great progress towards malaria elimination is possible through public-private partnerships in this context. Studies in endemic countries show that the private sector is a key player in the fight against malaria. The median proportion of children under 5 years receiving suspected malaria care from the private sector in SSA was 33.2%, as per a household survey running between 2017 and 2023 (14). In Sri Lanka, a partnership between the Ministry of Health and a private sector partner helped the country to be certified by the WHO as “malaria-free” in 2016 (15). This was seven years after the end of a 30-year-old separatist conflict in some regions. In Tanzania and Ethiopia, PPPs for malaria case management and distribution of mosquito nets were effective in providing health services to vulnerable populations (16, 17). The Zambian Malaria Control partnership with an agribusiness company increased insecticide treated nets coverage in hard-to-reach rural areas. They leveraged the company's infrastructure and capacity to supply farming inputs to the target population to reduce the costs of the exercise and ensure at least 95% coverage (18).

The feasibility of such a model should be harnessed to enable the effective implementation of the WHO Operational Framework in SSA. A literature review is needed to fill gaps and inform stakeholders on lessons from past PPPs at the climate change-malaria nexus. There is generally a dearth of literature on PPPs addressing health systems, most focus on filling gaps in the public healthcare systems. It is also noted that public-private partnerships are complex, involving organizations with different sets of goals, priorities, and plans. Literature shows that the PPPs face challenges and barriers that must be understood to avoid missed collaboration opportunities. Given that PPPs are context-specific, this rapid review will assess the challenges and opportunities for public-private partnerships in the health sector to pave the way for an efficient implementation of the WHO Operational Framework for Building Climate Resilient Health Systems in SSA.

## 2 Methods

### 2.1 Study design

This review was undertaken according to the Preferred Reporting Items for Systematic Reviews and Meta-Analyses (PRISMA) and the Centre for Reviews and Dissemination (CRD) guidelines (19, 20). The protocol was registered at the International Prospective Register of Systematic Reviews (PROSPERO) database with approval ID CRD42024617756.

### 2.2 Search strategies

A systematic literature search was conducted primarily in PubMed and Google Scholar databases. We also used AJOL as a supplementary tool to identify additional relevant studies that the primary databases might have missed. A search strategy was formulated using BOOLEAN operators “AND” and “OR” for all possible combinations of the following search terms: public-private sector partnerships, public health, and Sub-Saharan Africa. Supplementary Tables 1, 2 give more details on the applied search strategy.

### 2.3 Inclusion criteria

We considered all primary research articles detailing the challenges, barriers, and opportunities in public-private partnerships for improved health in Sub-Saharan Africa. We only included studies published since January 2010 till date and in the English language.

### 2.4 Exclusion criteria

We excluded studies that addressed PPPs in specific diseases other than malaria or did not provide information related to public-private partnerships. Studies conducted outside of SSA, those outside the date restriction. We also excluded literature reviews, conference abstracts and pre-prints.

### 2.5 Data screening and selections

We selected studies in two stages after the initial removal of duplicates. First, the titles and abstracts of the retrieved articles were screened for relevance by two independent reviewers, MM and SK. The full texts of potentially relevant studies were further assessed for data extraction. We used Mendeley for reference management of the potentially relevant articles. Disagreements were resolved through discussion with a third reviewer, JK. Subsequently, the full-text articles of potentially eligible studies were assessed by the same reviewers. Additionally, we manually searched the reference lists of the included publications to identify any additional relevant studies that may have been missed in the electronic search. We ensured internal consistency by training the reviewers on the criteria and process.

### 2.6 Data extraction

MM and SK extracted data from the selected studies using a standardized data extraction form, and any disagreements were resolved by consensus. The data extracted included author and year of publication, study site, research method, study setting, challenge/barrier, opportunity, and theme.

### 2.7 Study quality appraisal

MM and SK assessed the potential for bias in the eligible articles based on the Crowe Critical Appraisal Tool (CCAT). This tool was employed due to its validity for diverse research designs, a key characteristic of the papers included (21). We followed the procedure conducted by Crowe, the developer of the tool (22), appraising and scoring each of eight domain elements within a range of 0–5. Both reviewers participated in a calibration exercise, independently scoring three randomly selected studies and discussing results to harmonize interpretation of the CCAT criteria. Each included study was then independently appraised, with the reviewers blinded to each other's scores. We calculated the intraclass correlation coefficient to measure absolute agreement between the two raters. Any discrepancies in either domain specific scores or total scores were resolved through discussion to get consensus. We quantified inter rater reliability using the intra class correlation coefficient (ICC), which was 0.86 showing good agreement. A detailed table of domain-specific scores for all included studies is included in the supplementary files.

## 3 Results

### 3.1 Study selection

**Figure 1:**
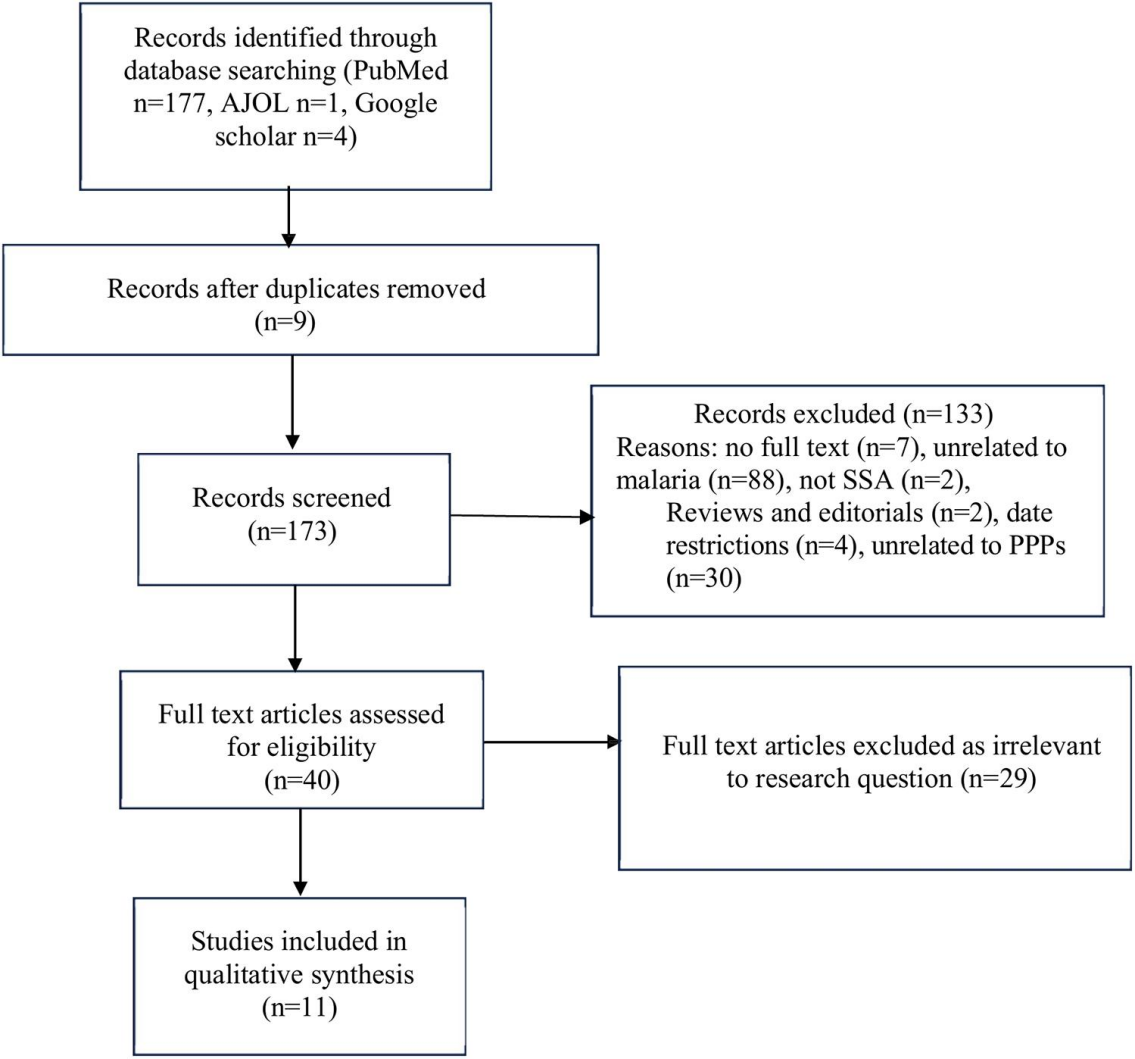
PRISMA flowchart.

We identified 173 research papers from our database searches, as summarized in Figure 1. Abstract and title screening resulted in the exclusion of 133 studies. The remaining 40 studies underwent full-text screening, 29 of which were excluded due to irrelevant study objectives or were secondary data sources such as reviews. This systematic review includes 11 articles focusing on the aim of this study. Figure 1 details the study selection process.

### 3.2 Characteristics of the studies

The data extracted from the included studies are detailed in Table 1. Five of the 11 studies were case studies of PPPs implemented in SSA. Four were cross-sectional surveys, and one was a media content analysis. There was also one randomized controlled trial.

**Table 1:** Included studies and their extracted data.

Author/s, year	Study site/s	Research method	Setting	Study Aim	Challenge/Barrier	Opportunity	Theme
Optimizing Investments in Malaria Treatment and Diagnosis [Cohen JM et al., (28)]	SSA	Household surveys	43 malaria endemic countries in SSA	To better understand the volume of antimalarials distributed through the private sector, the importance of the private sector in treating febrile disease, and the number of malaria infections reached by these drugs for better targeting of antimalarials	International donations for malaria control have decline (in 2012) for first time. The drugs in the private sector are predominantly affordable but ineffective monotherapies, reducing the effectiveness of the drugs.	Proper resource targetting and maximising their effectiveness. Private sector ACT subsidies, to improve access to effective drugs and overcome the challenge of fewer diagnostic kits. Especially in areas with high prevalence of malaria in patients presenting with fever. This is for the countries with high plasmodium positive results and high importance of private sector in treatment. For those with low fraction of ACT recipients estimated to be Pf+, but high importance of private sector in treatment its more cost effective to incerase access to diagnostic kits.	- Essential Medicines and Technology
Strengthening capacities and resource allocation for co-production of health research in low and middle income countries. Agyepong et al., (29)	ECOWAS, West Africa	Qualitative: workshops	6 Countries part of ECOWAS (Burkina Faso, Sierra Leone, Senegal, Cote d'Ivoire and Ghana)	Shared experiences and ideas for capacity strengthening and resource allocation for health research co-production in LMICs	Co-production of health research has been negelected in LMICs because of capacity and funding challenges. Challenges—1. Effectiveness while working with diverse knowledge and expertise (overcome through a capacity building exercise that enables the team to recognize the value of the different skills in the process of co-production), challenge 2. Power dynamics in the co-production environment (Overcome by creating a relaxed open discussion about power dynamics, led by a skilled facilitator. 3. Reliance on external donors for funding, when co-production is not a priority		Climate health research
African-led health research and capacity building- is it working? (Victoria O Kasprowicz et al., 2020)	SSA	Case study of SANTHE, an African led research consortium for HIV &TB	Capacity building approaches and SANTHE consortium case study	Discussed the challenges and approaches to health research capacity strengthening in Sub-Saharan Africa and propose that the recent shift to an African-led approach is the most optimal.	Lack of opportunities to meaningfully engage with peers and experts in the different fields of research 2. Difficulty in identifying large numbers of high-quality trainees for available positions 3. University level bureaucracy 4. Insufficient funding for research projects 5. poor training at school and undergraduate level,causing pipeline issues for high-quality trainee recruitment.		Health and climate research
Evaluation of the partnership between international non-governmental organizations and the State in the health sector in Mozambique (Munyangaju et al., 2021)	Mozambique	Cross-sectional study through standardized, semi-structured questionnaires	Investigation of the interactions between the MOH and International NGOs	Described the partnership between the national health system (public sector) and the INGOs in the health sector from the perspective of the healthcare workers.	1. internal migration of MOH workers to the INGOs especially due to wage disparities, thus weakening the health systems		Health Workforce
The South African Competition Commission COVID-19 easing of competition rules for private healthcare to facilitate public-private interaction—a media content analysis [Abdullah et al., (25)]	South Africa	Media Content Analysis	Online and social media article on the block exemption for healthcare to adapt competition regulations	Investigated how a change in competition regulation of private healthcare during a state of emergency was communicated in the media.	Communication gap between the government and the media. Inadequate media reports about government initiatives to enhance PPPs; no direct communication with the public on how the initiatives would benefit them, vital information about the exemption practices were not communicated *via* the media.	Inclusion of journalists on national emergency teams, and the development of guideline assisting both governments and journalists communicate during emergencies	Integrated Risk Monitoring and Early Warning, Health Workforce
Improving rational use of ACTs through diagnosis-dependent subsidies: Evidence from a cluster-randomized controlled trial in western Kenya (O'Meara et al., 2018)	Kenya	Randomized Controlled Trial	Private retail outlets and exisiting community health worker programs in diagnosis and subsequent dispensing of ACTs	Evaluated the public health impact of an innovative strategy that targets ACT subsidies to confirmed malaria cases by coupling free diagnostic testing with a diagnosis-dependent ACT subsidy		Circumventing complex regulatory strategies to target the delivery of antimalaria drugs through interventions that influence individual behaviour, in the context of heavily subsidized ACTs in the retail sector to curb overconsumption.	Leadership and governance, essential medicines and technologies
Expanding access to maternal, newborn and primary healthcare services through private-community-government partnership clinic models in rural Kenya: the Ubuntu-Afya kiosk model (Gatakaa et al., (30))	Kenya	Cross-sectional study through baseline and endline surveys	Evaluation of a network of 16 self-sustaining community medical centers (Ubuntu-Afya Kiosk) in rural areas	Examined the effect of a PPP approach of community medical centres on MNH access in Homa Bay, over a 2 year period of intervention.		Development of community medical centers, wiht a co-ownership model with beneficiary communities as self-help groups in partnership wiht county governments. The self-help group help with overseeing the operations of the medical centres, essentially enhancing social responsibility, supporting business endurance, securing market loyalty and helping to navigate socio-political challenges. The County Governments provide human resource and universal health commodity support, essentially enhancing sustainability of the interventions.	Emergency preparedness and management
“Get us partnerships!”—a qualitative study of Angolan and Mozambican health academics' experiences with North/South partnerships [Craveiro et al., (26)]	Angola, Mozambique	Qualitative Study on UDI-A using semi structured interviews and focus group discussions	University Development and Innovation—Africa project (UDI-A) is coordinated by NOVA University of Lisbon (P1), involving partners in Angola and Mozambique—Universidade Agostinho Neto (P5)-, Universidade Katyavala Bwila (P7), Universidade Eduardo Mondlane (P6), Universidade do Lúrio (P8), and Europe—Kings College London (P2), Maastricht University (P4), Université Libre Bruxelles (P3)	Examined potential outcomes of a collaboration with European institutions on participants' academic pathways, investigating the conflict between different imaginaries of capacity-building and partnerships, focusing on how Angolan and Mozambican health sciences researchers experience international collaborations.	1. Research assymetries between Europe and Africa, in aspects such as established structures for research and research dissemination roadblocks. 2. Common for North/South partnerships for capcity building to not take local contexts into account, by not taking local stakeholders' inputs		Health and Climate research
Enhancing Formal Educational and In-Service Training Programs in Rural Rwanda [Cancedda et al., (31)]	Rwanda	Case Study	Partnership between Rwanda's Ministry of Health and U.S. NGO Partners in Health, Harvard Medical School and Brigham and Women's Hospital.	Described a partnership launched in 2005 by Rwanda's Ministry of Health with the U.S. nongovernmental organization Partners In Health and with Harvard Medical School and Brigham and Women's Hospital.		Long-term North–South academic partnerships that seek both to strengthen health service delivery by building health workforce capacity in host countries and to simultaneously expand faculty and student engagement in global health within donor institutions.	Health Workforce
Leveraging massive open online courses to expand quality of healthcare education to health practitioners in Rwanda	Rwanda	Case Study	Partnership between Rwanda Ministry of Health and Harvard Initiative on Global Health Quality (HIGHQ) to launch a Massive Open Online Course	Summarised an innovative educational partnership between Harvard Initiative on Global Health Quality and Rwanda, to develop a blended-learning model to bolster participation in this new course among Rwandan healthcare professionals		Expansion of educational opportunities for healthcare providers and policy makers through open access courses that provide an overview of climate resilience for a global audience	Health Workforce
Challenges in health service delivery under public-private partnership in Tanzania: stakeholders' views from Dar es Salaam region (Nuhu et al., 2020)	Tanzania	Qualitative Case Study	Stakeholders, views and experiences in Kinondoni Municipality, Dar es Salaam	Investigated challenges encountered in implementing public-private partnership institutional arrangements in health service delivery in Kinondoni Municipality, Dar es Salaam, Tanzania.	1. Regulatory issues—inadequate guidelines to address emerging challenges such as bureaucracy in government mechanisms and a lack of skilled personnel to oversee PPP arrangements. 2. Inadequate resources—delays in disbursments especially by the government delays the provision of services 3. Lack of trust between PPP partners—a lack of fulfilment of decisions agreed upon breeds a lack of trust between the partners, as well as a lack of representation in the decision making bodies. 4. Ineffective monitoring and evaluation of the agreed performance indicators 5. insufficient consultation and communication—representatives from the private sectors not being consulted		Leadership and governance

### 3.3 Synthesis of challenges faced by and opportunities for PPPs in the implementation of the WHO operational framework

The studies show that the most challenges were relevant to leadership and governance, health workforce, health financing, and climate and health research. On the other hand, opportunities lie within the health workforce, essential medicines and technologies, and emergency preparedness and management. We employed a narrative synthesis guided by the WHO Operational Framework for Building Climate Resilient Health Systems to analyze identified challenges and opportunities.

#### 3.3.1 Challenges

In *leadership and governance,* inadequate and ineffective legal and policy frameworks hinder the effectiveness of PPPs. In Tanzania, Local Government Authorities (LGA) were contracted for personnel capacity building, but implementation was ineffective despite allocated budgets. Instead, implementation activities were overseen by the Ministry of Health so that LGA partners were answerable to the highest office—leading to bureaucratic governance issues. At the same time, the private partners would not adhere to regulations instituted by the municipality. Government decision-making excluded private partners due to a top-down approach and a lack of transparency. This led to limited buy-in and adherence among private partners (23). Power dynamics between governments and private partners should be considered carefully. The private health facilities in this study felt overpowered because they were under-represented in the decision-making bodies. They didn't have enough influence over decisions made and lost trust in the goodwill of the public sector, making the partnership unsuccessful.

We also noted challenges in the *health workforce* component of PPPs. A study evaluating State-NGO partnerships for health in Mozambique found that the wage disparities between the Ministry of Health and International Non-governmental Organizations (INGOs) led to the demotivation of health workers in the MOH, migration of workers from the MOH to the INGOs and conflicts between the different sets of health workers. As a result, the human resource challenge in the health system in the country was exacerbated (24). This component requires institutional capacity development by including the community through effective communication through the media. However, a media content analysis in South Africa exposed a communication gap between the government and the media. This resulted in inadequate media reports about government actions to enhance PPP activity and how the activities would benefit the public. This also hampers integrated risk monitoring and early warning, where timely warnings should be communicated to and through the media for effective action to prevent adverse health outcomes (25).

From the literature included, we found a lack of systemic integration of climate risk assessments into health planning under the component of *Vulnerability, capacity and adaptation assessment.* These assessments require and deepen an understanding of the populations at risk and the most appropriate health system responses. The exclusion of southern health academics in global North-South partnerships from agenda setting and high-level decision-making processes (26) may lead to assessments that overlook locally relevant indicators. This would result in inappropriate adaptation strategies, limiting the effectiveness of global North-South partnerships for vulnerability assessments. This challenge is also detailed in the paper by Kasprowicz (27), where the dependence of African institutions on external funding limits them to donor-driven research resulting in studies that are poorly aligned with local health system needs. A lack of African leadership hampers the generation and application of the contextual knowledge necessary for adaptation responses.In *essential medicines and technology*, the availability of predominantly affordable but ineffective monotherapies in private-sector drug outlets undermines optimized investments in Artemisinin Combination Therapy through PPPs. In addition, non-malarial febrile illnesses are treated with anti-malarial drugs since private sector outlets sell the drugs without confirmatory diagnosis (28). However, the place of private-sector outlets in malaria diagnosis and treatment is significant. As detailed in the introduction, the median proportion of children under 5 years receiving suspected malaria care from the private sector in SSA was 33.2%, according to a household survey from 2017–2023 (14).

Papers on partnerships in the *Health and climate research* component also exposed challenges. Co-production of research—collaboration with the primary beneficiaries of a study in designing, conducting, and interpreting research findings is ideal for contextualized decision-making. However, this approach has been neglected in Low Middle-Income Countries due to capacity and funding challenges (29). This is mainly because of an over-reliance on funding from external donors, whose priorities may not accommodate co-production research. A lack of opportunities for researchers to meaningfully engage with peers and experts restricts the exchange of ideas, information, and critical feedback on work, and the development of partnerships for African-led research projects. Consortiums in the continent with capacity-building initiatives also face difficulties in identifying sufficiently large numbers of high-quality trainees for available positions. This is attributed to poor training at school and undergraduate levels and sometimes university-level bureaucracy. Insufficient funding for research projects is also a challenge for south/south partnerships (27). As for north/south partnerships, the African financial context undermines research and development compared to the European context. Research dissemination systems are also undeveloped, leading to cases where researchers from the global north obtain data from Africa and publish articles without crediting the African counterparts. The lack of robust research structures in local universities plays a key role in limiting the reach of research conducted in Africa (26).

As for *Climate-Resilient and Sustainable Technologies and Infrastructure,* we noted the success of the Ubuntu-Afya Kiosks, which are solar-powered clinics developed in rural Kenya by Gatakaa and colleagues (30). One of the challenges noted in the paper is financial sustainability due to operational costs that may become burdensome to community cooperatives. At the same time, we didn't find any information on standardized climate-resilience criteria for infrastructure planning and evaluation. There is a lack of a minimum set of climate-proofing standards for different climate risks that would ideally be paired with local adaptation protocols, accounting for area specific risks and vulnerabilities.

#### 3.3.2 Opportunities

Despite the challenges in the papers reviewed, we also identified opportunities for public-private partnerships to implement the WHO framework.

In the *health workforce* component, greater collaboration would be facilitated by including journalists on national emergency teams. Furthermore, an opportunity for enhancing partnership lies in developing guidelines to assist communication between governments and journalists during emergencies for better information flow to the public to prevent adverse health outcomes (25). Long-term North-South partnerships where the global North medical schools and academia partners build local health workforce capacity through formal education and in-service training programs should be explored. The northern partners would have the incentive of opportunities for international deployment for their students to generate new knowledge in global health (31). Lastly, massive open online courses (MOOCs) in partnership with faculties in higher learning institutions to expand educational opportunities for healthcare providers in LMICs are an opportunity, as seen when the Rwandan Ministry of Health and Harvard University partnered for patient safety and quality of care training (32).

In *Essential Medicines and Technologies,* initiatives towards proper resource targeting in partnership with the private sector would maximize effectiveness. This includes subsidies to the private sector by governmental agencies depending on the priority need for either ACTs or rapid diagnostic kits to improve access and health outcomes. In areas of high plasmodium positive results and engagement of the private sector for malaria treatments, ACT subsidies would make more sense. On the other hand, in areas with low fractions of plasmodium-positive results among ACT recipients, subsidies for rapid diagnostic kits would increase the cost-effectiveness of programs and maintain the impact of malaria treatment activities (28). In areas with heavily subsidized ACTs in the retail sectors, behavior-influencing interventions for rational use of antimalarials would help bypass complex regulatory strategies. An example is the coupling of free diagnostic testing with diagnosis-dependent ACT subsidies implemented in Western Kenya (33).

An opportunity for *Emergency preparedness and management* lies in developing community medical centers with a co-ownership model, where beneficiary communities partner with county governments as self-help groups. The self-help groups help with overseeing the operations of the medical centers, essentially enhancing social responsibility in preventing and responding to health risks due to climate change, supporting business endurance, securing market loyalty, and helping to navigate socio-political challenges. The governments provide human resource and universal health commodity support, essentially enhancing the sustainability of interventions (30).

Even though our review found no PPP engagement in monitoring and forecasting of climate sensitive health risks, there are opportunities for partnerships with telecom private sectors for mobile alerts and surveillance. Given that the online MOOCs were delivered via mobile and internet platforms to reach remote practitioners, these platforms could be integrated with mobile alert systems for climate health communication (32). This partnership would strengthen *integrated risk monitoring and early warning* against climate-sensitive malaria risks.

Within the *climate and health research* component, PPPs between governments, researchers and implementing NGOs can leverage co-production mechanisms of research to drive evidence generation (29). This would ensure that research is relevant to policy and health system priorities, while ensuring that research is not donor driven or fragmented. Another opportunity would be to use partnerships to fund and partner with African research institutions for climate health studies led by local experts (27). This would add contextual depth and enhance the ownership of evidence base creation and increased long-term capacity among the institutions.

## 4 Discussion

We examined the challenges and opportunities for PPPs in implementing the WHO operational framework for climate-resilient health systems. No study provided information on health system-wide partnership activities, showing that most partnership activities directly affect only one or two of the ten components in the framework. Given the highly contextualized nature of PPPs, we avoided papers outside SSA and reporting on partnerships for improved outcomes in specific diseases other than malaria. Reported challenges fell into one of the following components—leadership and governance, essential medicines and technologies, health workforce, and health and climate research. Reported opportunities were in the health workforce, essential medicines and technology, and emergency preparedness and management.

Key lessons on partnerships show that the goals of the partnership should be in the interests of the public, and the governance structures should facilitate the interests of the private partners. There should also be a balance of bargaining power between the partners to alleviate issues related to control especially concerning budgets (34), with adequate consultation and representation of private partners in the planning and implementation stages of the PPPs (23). An alignment of the partners' priorities and interests would ensure no resistance to collaborations (13, 35). At the same time, the private sector partners can facilitate trust with the government by limiting the migration of healthcare workers from the public sector for higher pay (24).

Bureaucracy within the public sector has been found to diminish the incentive for the private sector to partner with governments (13). Time consuming administrative processes and structures almost cost the Tanzanian government a partnership on the National Voucher Scheme from bed-nets after a one-year delay before commencement of activities (36). This weak state of governance systems in the public sector led private partners, Global Health Initiatives in particular, to work through parallel structures for effective usage of funds (37).

Studies investigating contractual agreements between the partners (who had a formal contractual agreement) noted weaknesses in contract development, especially for performance monitoring guidelines and incentives in the public sector. This led to poor quality outcomes, as there were no clear guidelines for private partners linking payment and performance, and no incentives (10). This calls for greater attention from the public sector to PPP design processes and their capacity to manage partnerships. All partners should have access to incentives to drive active participation in line with the agreements (38).

Inclusive governance and research structures should be established to ensure that southern voices in North-South partnerships are not sidelined, for contextually relevant and effective climate adaptation responses. This is especially important to avoid limited local ownership. Another way to go about it is to step up in context research leadership through South-led research partnerships for stronger risk assessments and solutions. Other challenges include the lack of a minimum set of climate-proofing standards for different climate risks in the region for improved climate-resilent and sustainable technologies and infrastructure. Strict universal criteria may not be appropriate, but resilience must be structured to enable faster progress through benchmarking, policy coherence, and accountability. In addition, standard frameworks enable alignment with donors and governments when attracting investements in public-private partnerships.

At the root of a majority of the challenges we identified, is a lack of effective communication. This situation breeds mistrust and reduces the effectiveness of the partnerships regardless of the context (35, 39, 40). Effective communication channels will also enable effective accountability systems (23).

We also identified opportunities worth exploring, especially in the early stages of the implementation of the WHO Operational Framework in Sub-Saharan Africa. There is a need for large scale capacity building within the health workforce for climate resilience. The use of Massive Online Open Courses in partnership with Global North Institutions would facilitate the access of quality education at scale as has been done in Rwanda (32).

There are lessons to learn from PPPs outside the Sub-Saharan Africa context. In India, the Comprehensive Case Management Project—a partnership between the government, a local research institute and Medicines for Malaria Ventures led to a 47% decline of positive cases (41). This partnership and the intervention packages were premised on a detailed situational analysis that highlighted areas of weakness leading to reversed gains from previous control efforts. The partnership was effective and successful, showing the importance of operational research spearheaded by national control programs with the pre-requisite political will and technical support of local research organizations. The necessity for contextual modifications to programmatic intervention was also key to the success of another Indian PPP—the Malaria Elimination Demonstration Project. This partnership encountered challenges such as reluctance to adoption of digital applications for data collection and reporting, which was overcome by quality training (42). In addition, the partnership ensured the sustainability of replicability of their interventions by building up from existing state government systems and strategies. This alignment enabled the government to replicate the practices in other districts to good effect (43).

Another PPP in Sri Lanka for malaria elimination ensured that teams were recruited locally, even when lacking the technical expertise (15). The private sector partners invested heavily on capacity building and sustainable development to enable a smooth transition in the long term. However, at the executive level only highly skilled professionals were employed for managerial and scientific tasks. More importantly, the private partners maintained a close relationship with all governmental and non-governmental stakeholders. Initially, coordination of activities and amalgamation of data between the malaria control program and the private partners was strained as this was the first PPP the Ministry of Health was involved in. This was resolved effectively through a common monitoring and evaluation plan that guided the operations of both entities.

### 4.1 Limitations

We acknowledge that the findings reflect on PPPs specific to the unique contextual challenges in different geographical settings in the SSA region. The findings here may not universally apply to all countries within the region. In addition, given that PPPs targeting climate resilience in malaria control are at an early stage of development, information on essential components such as vulnerability assessments are not available within SSA. We have included lessons from countries outside SSA to highlight their importance for effective PPPs. We also acknowledge that the time constraints for this rapid review limited the number of databases searched therefore key papers may have been missed. This also includes the fact that we only included articles published in English and time-restricted from 2010 to date.

## 5 Conclusion

Despite the many challenges, PPPs remain a viable alternative in the adaptation of the WHO Operational Framework. This is particularly the case when the technical and financial capacities of the countries in the SSA region are considered. There are lessons to be derived and best practices to institute from case studies of previous partnerships, especially in malaria control. The design of the PPPs must engage private partners as equals with many strengths to leverage for improved outcomes in malaria control in the face of climate change. Strong communication channels should be put in place to avoid many of the challenges described, as we engage in new opportunities. We recommend the formation of PPPs between Ministries of Health, national research institutions, and private sector players for context-specific climate health studies. These include vulnerability, capacity and adaptation assessments, which should also leverage co-production modalities. The coordination of activities in these PPPs must be developed through consensus between the partners, and documented in monitoring and evaluation plans to reduce opportunities for operational bottlenecks.
